# Case report of bilateral penetrating renal trauma caused by a wooden stick

**DOI:** 10.1097/MD.0000000000019853

**Published:** 2020-04-17

**Authors:** Jing Xie, Ying Liu, Tong Chen, Ke-Feng Xiao

**Affiliations:** Department of Urology, Shenzhen People's Hospital, Second Clinical Medical College of Jinan University, Shenzhen, China.

**Keywords:** bilateral penetrating renal trauma, nephrectomy, renal salvage, renorrhaphy

## Abstract

**Rationale:**

Kidney is the most frequently injured organ of the genitourinary system during trauma. Bilateral penetrating renal trauma (BPRT) is extremely rare and sporadically reported in the previous literature. Here, we reported a unique case of BPRT.

**Patient concerns:**

A 43-year-old man, with no medical history, was accidentally penetrated by a wooden stick and presented with sharp pain in the left flank.

**Diagnosis:**

Laboratory tests revealed microscopic hematuria, mildly elevated leucocyte and amylase, normal hemoglobin (145 g/L) and creatinine (1.05 mg/dl). Computed tomography demonstrated bilateral penetrating renal injuries with perinephric/subcapsular hematoma, fracture of the second lumbar vertebra and 10th rib.

**Interventions:**

An emergency exploratory laparotomy was executed immediately. According to the American Association for the Surgery of Trauma Organ Injury Scale grading system, grade V and III injuries were considered for the left and right kidney, respectively. Nephrectomy and renorrhaphy were performed on the left and right kidney, respectively.

**Outcomes:**

The postoperative course was uneventful. Eleven days after the surgery, the patient discharged with no complications.

**Lessons:**

We present a rare and challenging case which was handled successfully, and it may provide useful information for the management of BPRT.

## 1 Introduction

Although located in relatively protected retroperitoneum, kidney is the most frequently injured organ of the genitourinary system during trauma. Renal injuries accounts for approximately 0.3% of all trauma according to the National Trauma Data Bank database.^[1]^ It occurs more common in the young population with an obvious male predominance. Compared to blunt trauma, penetrating trauma is less common, accounting for 20% to 39% of renal trauma cases.^[2,3]^

The primary mechanism of penetrating renal injury varies considerably among different regions. In the United States, gunshot wound accounts for approximately 72.2% of penetrating renal injuries.^[3]^ While in other countries such as United Kingdom and Canada, stabbing wound contributed to 87.3% and 88% of penetrating renal traumas, respectively.^[4,5]^ Bilateral renal trauma is infrequent in penetrating renal injuries and its management remains challenging, with the potential risk for complete loss of renal function. Here, we reported an interesting case of bilateral penetrating renal trauma (BPRT) in a 43-year-old man with no medical history. As far as we know, such unusual condition and associated management have not been previously reported.

## 2 Case report

**Figure 1:**
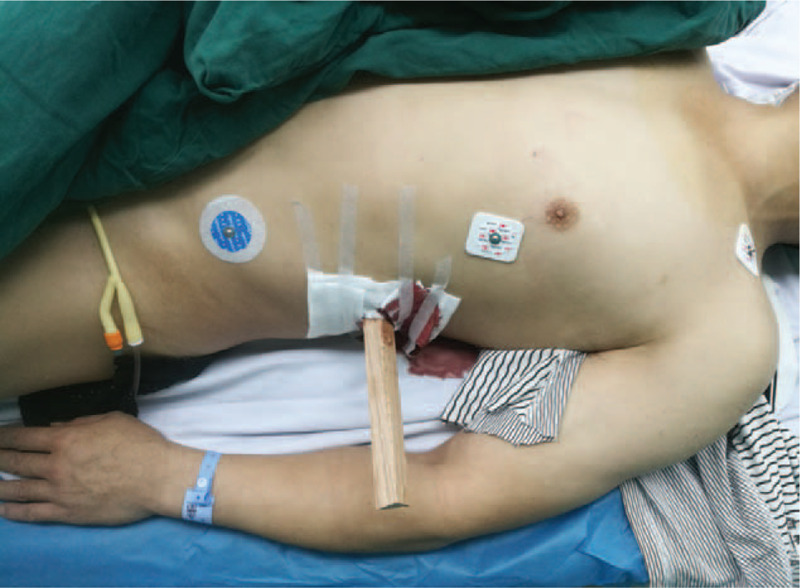
The patient was penetrated by a wooden stick in the left flank, with continuing blood leakage. No gross hematuria was observed in the catheter.

A 43-year-old man, working in a furniture company with no medical history, was accidentally penetrated by a wooden stick during his routine factory touring. He was immediately taken to the emergency department of the local hospital by ambulance. Feeling tearing anguish in the left flank, the patient was dysphoric and drenched in sweat. On physical examination, he was hemodynamically stable (blood pressure, 110/70 mmHg) and tachypneic (respiratory rate, 30 times/minute). There was a solitary entrance wound in the left flank, with a stick penetrated in and continuing blood leakage (Fig. 1). Urethral catheter was inserted and no gross hematuria was observed.

**Figure 2:**
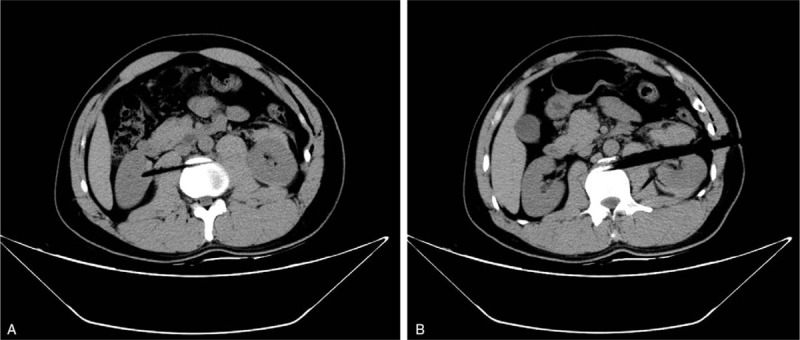
Preoperative CT scan (A: The stick went through the right psoas muscle and terminated in the right renal parenchyma. B: The stick went through the left kidney, the left psoas muscle and the anterior body of the second lumbar vertebra.)

Laboratory tests revealed microscopic hematuria, mildly elevated leucocyte and amylase, normal hemoglobin (145 g/L) and creatinine (1.05 mg/dl). Chest and abdomen computed tomography (CT) scan was performed in the local hospital, which demonstrated bilateral penetrating renal injuries with perinephric/subcapsular hematoma, fracture of the second lumbar vertebra and 10th rib. Neither pleural injury nor lung injury was observed. The stick went through the left kidney, the anterior body of the second lumbar vertebra and bilateral psoas muscle, terminating in the right renal parenchyma (Fig. 2). Fortunately, there was still a distance between the stick and ventral aorta and inferior vena cava. However, there was concern that any displacement of the wedge-shaped stick might cause life-threatening hemorrhage.

Owing to the intractability of this case, the patient was transported to our hospital. An emergency exploratory laparotomy with the midline abdominal incision was performed immediately. We used a self-retaining retractor to obtain satisfactory visibility. After clamping the left renal pedicle, we slowly pulled out the stick according to its penetrating path on CT scan. The hilum of the left kidney was transected, and the collecting system was seriously damaged and contaminated, with extensive parenchymal and vascular injuries. It was considered a grade V injury according to the American Association for the Surgery of Trauma (AAST) Organ Injury Scale grading system (Table 1). Since the left kidney was shattered beyond repair, we decided to excise it totally. Then we explored the right kidney and found an approximately 2.0 cm posterior laceration in the inferior pole, without urinary extravasation. It was deemed a grade III injury according to the AAST grading system. After washing out the sawdust and sterilizing the laceration with 0.5% iodophor, we meticulously performed renorrhaphy of the right kidney. Then the abdominal cavity was explored and no intestinal injury was found. The fracture of the second lumbar vertebra was handled by spine surgeons. Intraperitoneal drainages were routinely placed on both sides and the laceration of the left flank was repaired. During the operation, four units of packed red blood cells were transfused. Tetanus antitoxin and piperacillin and tazobactam were injected to prevent tetanus and bacterial infection. The patient was transferred to surgical intensive care unit for resuscitation.

**Table 1:**
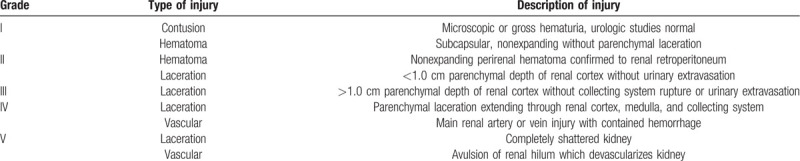
Renal trauma classification by the American Association for the Surgery of Trauma^[9]^.

The postoperative course was uneventful. There was approximately 2000 mL of urine for the next 24 hours postoperatively. Postoperative chest radiograph revealed slight exudation of bilateral lungs. Eleven days after the surgery, the patient recovered well and discharged with no complications. His discharge blood pressure was 120/76 mmHg and creatine was 1.50 mg/dl.

## 3 Discussion

For patients with suspected renal trauma, initial assessment including airway, breathing, and circulation is necessary. In cases of hemodynamic instability and severe hemorrhage, immediate exploration may be considered preferentially.^[2]^ However, If the patient is hemodynamically stable, a thorough evaluation including history collection, physical examination, laboratory tests and imaging should be performed.^[6]^ Patient history and the injury mechanism are very important for making the right treatment decisions, especially in cases of solitary kidney. Physical examination may help to assess the location, extent and severity of the trauma. Any sign indicating renal trauma should be noted, such as visible hematuria, rib fractures and flank/upper abdomen hematoma. Hematocrit and creatinine levels are necessary to evaluate current blood loss status and baseline renal function. Urine analysis is used to diagnose microscopic hematuria.

Currently, the gold standard imaging for hemodynamically stable patients with penetrating renal trauma is intravenous contrast-medium enhanced CT. It has replaced intravenous pyelography in virtue of its wide availability, superior anatomical and functional information and the ability to identify associated injuries. Although the contrast medium was reported to induce nephropathy in blunt trauma patients, the rate was low (4%) and its toxicity has not been confirmed in penetrating renal injury patients.^[7]^ The goal of initial imaging is to demonstrate contralateral kidney and potential renal abnormalities, accurately grade the renal injury, and identify the associated injuries. For penetrating renal trauma patients, the most frequent findings on CT were parenchymal disruption and perirenal hematoma. Reimaging is recommended for patients with high-grade injuries 2 to 4 days later, and patients with clinical signs of deterioration or postoperative complications, such as fever, persistent hematuria and ongoing blood loss.^[8]^ Renal trauma is most commonly classified according to AAST grading system, which is based on the extent of damage to the renal parenchymal, collecting system, and/or renal vasculature.^[9]^ The AAST classification was validated by several researches and paramount in treatment decision making. It was reported to be significantly associated with the need for surgical intervention and the risk for nephrectomy.^[10]^

Treatment options include conservative management, minimally invasive intervention, and open surgery.^[6]^ Conservative management typically involves bedrest, analgesia, hemodynamic monitoring, serial laboratory evaluation and reimaging when there is any deterioration. Minimally invasive intervention includes angioembolization for uncontrolled bleeding, or placement of ureteral stent, perinephric drain and nephrostomy tube for urinary extravasation. Open surgeries were generally nephrectomy, partial nephrectomy, renorrhaphy, renal packing, or autotransplantation. In the past few decades, the management of renal trauma has undergone a paradigm shift. Nonoperative management is currently the standard care for low-grade renal injuries, and is also recommended for high-grade renal injuries in hemodynamically stable patients.^[11]^ In a multicenter study, nonoperative management was performed in approximately 75% of grades IV and V renal injury patients, and only failed in 6.5% of those patients. Moreover, conservative management of high-grade renal injuries was not associated with prolonged hospital stay.^[12]^

Penetrating renal injuries frequently involve the renal vascular system, disrupt Gerota fascia, and limit the inherent mechanism of retroperitoneal tamponade, which may lead to an increase in rates of nonoperative failure. In a previous study analyzing the data of >9000 renal injuries, angioembolization was showed to be threefold likely to fail for penetrating renal injury compared with blunt renal trauma.^[13]^ Moreover, a significant risk of renovascular sequelae was observed in penetrating renal injury patients treated with nonoperative management.^[4]^ Patients with penetrating renal injuries were noted to have higher proportion of grade IV and V renal injuries, higher overall rate of concomitant injuries, angioembolization and nephrectomy (27% vs 7% for blunt). ^[5,10]^

BPRT is extremely rare and sporadically reported in the previous literature. The management of BPRT remains challenging, placing particular emphasis on renal parenchymal preservation. Schecter reported 3 BPRT cases in detail, which were all with unstable hemodynamics and managed with immediate laparotomy.^[14]^ Conservative management of stable perinephric hematomas discovered at laparotomy was recommended, particularly if the contralateral kidney required exploration for hemostasis. Optimal control of the ipsilateral renal vessels before mobilizing the kidney is important, because it permits rapid hemostasis and avoids unnecessary nephrectomy, thus increasing the chance of renal salvage. Synchronous IV and V bilateral penetrating renal injuries, with stable retroperitoneal hematoma, were reported to be successfully managed by angioembolization.^[15]^ In the unusual setting of devastating bilateral renal injuries or solitary kidney injury, renorrhaphy and vascular repair in situ or extracorporeal renorrhaphy with immediate autotransplantation may be considered as a last resort.^[16]^

In this case, contrast-enhanced CT was not performed because non-contrast enhanced CT had already been performed in the local hospital. We attempted to avoid excessive radiation exposure and carry out the emergency laparotomy immediately. Exploratory laparotomy was mandatory, because the stick as a foreign body must be taken out and the left kidney was seriously damaged. Renal exploration was done via a transperitoneal approach with early control of the renal pedicle, which enabled us to thoroughly evaluate the retroperitoneal area. Nephrectomy and renorrhaphy were respectively performed on the left and right kidney, according to the severity of renal injuries. Close monitoring is imperative after the surgery. We recommend that postoperative hospital stay should be at least one week, since nonnegligible complications such as arterial pseudoaneurysms or arteriovenous fistula may occur 6 to 8 days after penetrating renal injury.^[4]^ In summary, we present a rare and challenging case which was handled successfully, and it may provide useful information for the management of BPRT.

## 4 Consent

Written informed consent was obtained from the patient before and after all procedures, and for the publication of this paper.

## Author contributions

**Conceptualization:** Ke-Feng Xiao.

**Data curation:** Jing Xie, Ying Liu.

**Investigation:** Jing Xie, Tong Chen.

**Project administration:** Ke-Feng Xiao.

**Resources:** Ying Liu, Tong Chen.

**Writing – original draft:** Jing Xie.

**Writing – review & editing:** Ying Liu, Tong Chen, Ke-Feng Xiao.
